# Suboptimal Iodine Status among Pregnant Women in the Oslo Area, Norway

**DOI:** 10.3390/nu10030280

**Published:** 2018-02-28

**Authors:** Sigrun Henjum, Inger Aakre, Anne Marie Lilleengen, Lisa Garnweidner-Holme, Sandra Borthne, Zada Pajalic, Ellen Blix, Elin Lovise Folven Gjengedal, Anne Lise Brantsæter

**Affiliations:** 1 Department of Nursing and Health Promotion, Faculty of Health Sciences, OsloMet — Oslo Metropolitan University, Oslo 0310, Norway; inger.aakre@hioa.no (I.A.); annemarie.lilleengen@hioa.no (A.M.L.); lisa.garnweidner-holme@hioa.no (L.G.-H.); sborthne@gmail.com (S.B.); Zada.Pajalic@hioa.no (Z.P.); Ellen.Blix@hioa.no (E.B.); 2 Faculty of Environmental Sciences and Natural Resource Management, Norwegian University of Life Sciences, Aas 1433, Norway; elin.gjengedal@nmbu.no; 3 Division of Infection Control and Environmental Health, Norwegian Institute of Public Health, Oslo 0403, Norway; AnneLise.Brantsaeter@fhi.no

**Keywords:** iodine status, pregnancy, iodine deficiency, iodine intake, urinary iodine concentration, urinary iodine excretion, Norway

## Abstract

Norway has been considered iodine replete for decades; however, recent studies indicate reemergence of inadequate iodine status in different population groups. We assessed iodine status in pregnant women based on urinary iodine concentration (UIC), urinary iodine excretion (UIE), and iodine intake from food and supplements. In 804 pregnant women, 24-h iodine intakes from iodine-rich foods and iodine-containing supplements were calculated. In 777 women, iodine concentration was measured in spot urine samples by inductively coupled plasma/mass spectrometry (ICP-MS). In addition, 49 of the women collected a 24-h urine sample for assessment of UIE and iodine intake from food frequency questionnaire (FFQ). Median UIC was 92 µg/L. Fifty-five percent had a calculated iodine intake below estimated average requirement (EAR) (160 µg/day). Iodine intake from food alone did not provide the amount of iodine required to meet maternal and fetal needs during pregnancy. In multiple regression models, hypothyroidism, supplemental iodine and maternal age were positively associated with UIC, while gestational age and smoking were negatively associated, explaining 11% of the variance. This study clearly shows that pregnant women in the Oslo area are mild to moderate iodine deficient and public health strategies are needed to improve and secure adequate iodine status.

## 1. Introduction

Iodine is an essential micronutrient for the synthesis of thyroid hormones that are critical for brain development and growth during fetal life and infancy. Iodine deficiency during pregnancy may affect cognitive function of the offspring and lead to mental impairment [1,2,3,4]. World Health Organization (WHO) considers iodine deficiency to be the single most important preventable cause of brain damage worldwide [5]. Universal salt iodization is the first-line strategy for the elimination of severe iodine deficiency; however, in Norway, no public strategy to ensure adequate iodine status currently exists. Because of successful programs of universal salt iodization in former severely iodine-deficient regions around the world, public health concern has shifted toward mild to moderate iodine deficiency, which remains prevalent in many regions, especially among pregnant women [6]. Iodine deficiency is often thought to be a problem in developing countries, however industrialized countries are not immune [7,8]. Indeed, concern is emerging that mild iodine deficiency might be prevalent in Norway, even though Norway has been considered iodine replete for six decades [9]. Results from the Norwegian Mother and Child Cohort Study (MoBa) have shown that a high proportion of pregnant women recruited from 2002 to 2008 had suboptimal iodine intake [10]. A recent study found that lactating women in Norway had low breast milk iodine concentration, and ¾ of the women had suboptimal iodine status [11]. In Norway, the most important iodine sources are milk and dairy products, due to iodine fortification of cattle fodder, in addition to seafood. The consumption of milk, yoghurt, and lean fish has declined over the past decades in some population groups. Only one brand of table salt is fortified with iodine and the permitted level (5 µg/g) is too low to affect iodine intake [9]. Iodine in drinking water is low (<0.2 µg/L) [9]. A report on iodine status in Norway highlighted the need for updated studies on iodine status among pregnant women [12]. The main objective in this study was therefore to evaluate iodine status by assessing urinary iodine concentration (UIC) and calculate iodine intake in pregnant women in the Oslo area, Norway. In addition, in a sub-sample, urinary iodine excretion (UIE) from 24-h urine samples was analyzed to compare the agreement between UIC and UIE and calculated iodine intake.

## 2. Materials and Methods 

### 2.1. Population and Study Design

Convenience sampling was used to recruit pregnant women from eight different Mother and Child Health Centers (MCHCs) in the Oslo area, Norway. The data were collected in two steps: first, pregnant women were recruited by midwives at MCHCs during antenatal care visits in the period from February to December 2016. All pregnant women who could read and write Norwegian were invited to participate. To assess iodine concentration at population level, at least 500 urine samples are required [13]. Around 50–100 women from each of the eight healthcare centers were asked to participate, in total 804 pregnant women were included. The women answered questions on background information (age, week of pregnancy, previous pregnancies, pre-pregnancy height and weight, educational level and smoking habits, country of birth, years in Norway and languages) and gave a spot urine sample (*n* = 777). In addition, iodine intakes from iodine-rich foods and iodine-containing supplements were calculated for the last 24-h (*n* = 804). Secondly, in a sub-sample of 49 of the 777 pregnant women, urinary iodine excretion was analyzed in 24-h urine, in addition to the spot urine samples. To provide information on habitual intake, they were asked to fill in a food frequency questionnaire with 31 food items in addition to a more in-depth 24-h dietary recall interview covering total food intake on the day of the 24-h urine collection. In total, 777 of 804 women delivered spot urine samples, of which 728 were in the main study and 49 in the sub-study. In total, 804 gave information on iodine intake, of which 755 were in the main study and 49 in the sub-study. 

After delivery of urine samples, all women received information sheets about dietary sources of iodine and recommendations for iodine intake. The women were informed about the study purpose and those willing to participate gave informed written consent. The present study was conducted according to the guidelines in the Declaration of Helsinki and was approved by the Regional Committee for Medical and Health Research Ethics Norway (2015/1845).

### 2.2. Urinary Iodine Concentration

Participants donated a non-fasting (random) spot urine sample in a labelled 100 mL Vacuette^®^ Urine beaker (Greiner Bio-One, Kremsmünster, Austria). Participants in the sub-study were given one 0.5 L and two 1 L high-density polyethylene plastic bottles (VWR International AS, Oslo, Norway) with wide opening and screw capped double lids for collecting one spot morning urine and 24-h urine. They were instructed to discard the first morning urine void on the day of urine collection, and collect all urine for the next 24 h. The morning urine void on the second day was to be collected in a separate container marked “morning”. The morning and the 24-h urine samples were stored at <4 °C in the time prior to handling. Aliquots of 1.00 mL morning urine were transferred into 15 mL, polypropylene (pp) centrifuge tube (Sarstedt, Nümbrecht, Germany) by means of a 100–5000 μL electronic pipette (Biohit, Helsinki, Finland) for determination of UIC and the rest added to the 24-h collection. All samples were stored refrigerated from the time of sampling until transportation to the laboratory. At the laboratory, the non-fasting spot urine sample where stored at minus 80 °C until analysis. Considering both the non-fasting spot urine sample, the morning and the 24-h urine samples, aliquots of 1.00 mL were diluted ten times with an alkaline solution (BENT), containing 4% (w/V) 1-Butanol, 0.1% (w/V) H_4_EDTA, 2% (w/V) NH_4_OH, and 0.1% (w/V) Triton X-100. Reagents of analytical grade or better and deionized water (>18 MΩ) were used throughout. The quantification of iodine was performed by means of the Agilent 8800 Triple Quadrupole ICPMS (Agilent, Santa Clara, CA, USA) using oxygen reaction mode. Iodine was determined on mass 127. Tellurium (Te) was used as internal standard. Certified reference materials (CRM) used for quality control was Trace Elements Urine L-1 (78 µg/L) and Trace Elements Urine L-2 (280 µg/L) from Seronorm. All the measured values of CRM were within the certified range. The same procedure was followed for blank samples as other samples, and all had values under the limit of detection (LOD, 0.4 µg/L) or limit of quantification (LOQ, 1.2 µg/L). The detection and quantification limits were calculated at three and ten times the standard deviation (SD) of blank samples, respectively. Intermediate precision (within-laboratory reproducibility) was <4%. The Norwegian University of Life Sciences in Aas (Faculty of Environmental Sciences and Natural Resource Management) performed measurement of the iodine concentration. In sub-study-participants, UIE was calculated by multiplying UIC in the 24-h urine and the 24-h urine volume;

UIE (μg/24 h) = UIC (μg/L) × Total urinary volume (L/24 h).(1)

Estimated iodine intake from UIE was calculated by the assumption that 90% of dietary iodine is excreted in urine (UIE µg/24 h × 100/90) [14].

### 2.3. Assessment of Iodine from Food and Dietary Supplements 

Iodine intakes from iodine-rich foods and iodine-containing supplements were calculated for the last 24 h. Most participants (*n* = 755) reported intake of iodine-rich foods, while women in the sub-study reported total food intake during the last 24-h and habitual intake (frequency) of selected food items. In the main study, the questionnaire included questions about intake of milk and yoghurt (number of glasses), cheese, eggs (including dishes), and fish (lean and fatty fish for dinner and/or on bread). These are the main food items contributing to iodine intake in Norway [10]. The reported food intakes were multiplied with the iodine concentration for each specified food to obtain iodine amount. In the sub-study (*n* = 49), iodine intake for the last 24 h was assessed by a 24-h recall performed as a semi-structured interview. The 24-h recall included all food and drinks consumed, not only iodine-rich foods as in the main study. All food items reported in the 24-h recall were manually coded and FoodCalc [15] was used to calculate food and nutrient intakes. In addition, sub-study participants reported their habitual food intake by answering 31 questions about average intake of selected food items/dishes, including eight questions about iodine-containing foods. Of these, three questions assessed intake of milk and dairy products, four assessed intake of fish and fish-dishes, and one assessed intake of egg and egg-dishes. The questions had seven answer alternatives, ranging from rarely/never to five times daily or more. The answers to the questions related to intake of milk, cheese, fish, and egg were converted to daily amounts and multiplied with the iodine concentration for each food item/dish. In all calculations, iodine concentrations reported in the Norwegian Food Composition Table [16] were used for all items except milk and egg. We applied 13 µg/100 g for milk and 30 µg/100 g for eggs, as analytical results for iodine concentration in these items were lower than the values in the food composition table [17,18]. For estimating 24-h iodine intake in the main study and the habitual iodine intake in the sub-study, we applied recipes to derive an iodine concentration in composite dishes and for averaging concentrations from different fish species. To account for iodine contributed by foods and dishes not covered by the assessed food items, we added 30 µg/day to each estimated total intake. The questionnaire filled in by all participants included a question about use of dietary supplements. Participants were asked to report each supplement by name and how many times weekly the supplement was taken. Using information provided by producers and labels, we calculated the daily amount of iodine contributed by iodine containing supplements and added this to the calculated 24-h and habitual intakes from food. 

### 2.4. Definitions of Iodine Status and Recommendations for Iodine Intake

In this study, we apply the epidemiological criteria’s for assessment of iodine nutrition established by WHO [5]. The recommended indicator for evaluation of iodine status is the population median UIC, and median UIC below 150 µg/L is considered to reflect insufficient iodine intake in pregnant women, while a median UIC between 150 and 249 reflects adequate iodine nutrition. The Institute of Medicine established an estimated average requirement (EAR) for iodine of 160 µg/day during pregnancy [19]. WHO recommends a daily iodine intake of 250 µg/day during pregnancy, while the recommended iodine intake in the Nordic countries is set to 175 µg/day during pregnancy [20], and the recommended daily intake for non-pregnant women of reproductive age is similar to the WHO recommendation of 150 µg/day [21].

### 2.5. Statistical Methods

Normally distributed data were presented as mean ± SD. Non-normally distributed data were presented as median and 25th–75th percentiles (p25–p75). UIC, UIE, short-term iodine intake from food and supplements, and habitual iodine intake were checked for normality using Q-Q plots and the Shapiro-Wilk test. Due to the skewed distribution, non-parametric tests were used. Independent group differences were examined using Mann-Whitney U-test and dependent group differences were examined using Wilcoxon’s signed rank test. Spearman’s rank correlation was used to evaluate the linear relationship between continuous variables. The UIC was used as dependent variable in multiple linear regression analyses. Because of skewed distribution, this dependent variable was log-transformed (lnUIC). All socioeconomic variables and iodine intake were selected to find candidate variables for inclusion in the model. After linear association (*p <* 0.10), the following variables were included in the initial model: maternal age, gestational age, smoking, hypothyroidism and mean daily use of iodine-containing supplement. All covariates showing a linear association (*p* < 0.10) in the crude regression models were included in a preliminary multiple regression model. Excluded variables were reintroduced and those that were still significantly associated in this model (*p* < 0.10) were retained in the final model. Analysis of the residuals was performed to examine the fit of the model. 

## 3. Results

Sample characteristics of the pregnant women are presented in Table 1. In the total sample, 53% of the women were in the third trimester of pregnancy, 77% were born in Norway and 53% expected their first child. The proportion of nulliparous women were higher in the sub-study (86%) than in the main study (52%). Women in the sub-study were more often highly educated (55%), had Norwegian origin (84%) and reported more often use of iodine supplements (45%) than women in the main study (32%). The iodine level in the supplements ranged from 150–200 µg.

Descriptive data of UIC and UIE are presented in Table 2. Median (p25–p75) UIC was 92 (59–140) µg/L. Median (p25–p75) UIC in first, second and third trimester were 92 µg/L (43–173), 96 µg/L (64–140) and 91 µg/L (58–130), respectively. No significant differences were found in UIC between the trimesters. Median (p25–p75) UIC in the 24-h urine collection was 91 (61–190) µg/L. Median (p24–p75) UIE was 120 (83–181) µg/L. 

**Figure 1:**
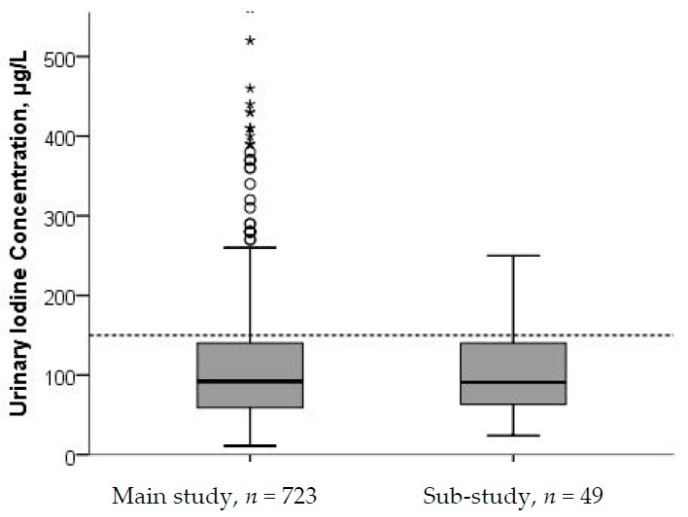
Urinary iodine concentration (UIC) from main study (*n* = 723) and sub-study (*n* = 49). Five cases with UIC > 500 µg/L in the main study were excluded from the figure. The stippled horizontal line marks the epidemiological criteria for assessing adequate iodine nutrition based on median UIC by the WHO [5].

As seen from the box plot in Figure 1, the median UIC and upper quartiles were below the recommended level of 150 µg/L in the main study and as well as in the 24-h urine in the sub-study. 

Calculated iodine intakes are shown in Table 3. In the main study, the median (p25–p75) 24-h iodine intake from food was 110 (70–150) μg/day. Including iodine from supplements, the median (p25–p75) iodine intake increased to 148 (88–251) μg/day. In the sub-study, the median (p25–p75) iodine intake by 24-h recall was 114 (78–149) μg/day from food and 143 (101–289) μg/day including iodine from supplements. The median (p25–p75) habitual iodine intake from food was 117 (95–147) μg/day and 149 (109–268) μg/day including supplemental iodine. The median amount of iodine contributed by supplements was 175 µg/day in iodine-containing supplement users in the main study and 150 µg/day iodine supplement users in the sub-study.

In the main study, 55% had a calculated iodine intake below EAR (160 µg/day). In the sub-study, 53% and 55% had calculated iodine intake below 160 µg/day in the 24-h recall interview and habitual intake, respectively. In the total sample, total iodine intake correlated with UIC; *r* = 0.26 (95% confidence interval (CI): 0.19, 0.32). In the sub-sample, total iodine intake correlated with UIE for the 24-h recall (*r* = 0.46; 95% CI: 0.21, 0.66) and for habitual food intake (*r* = 0.36; 95% CI: 0.09, 0.58). Significant, but slightly weaker correlations were found for the calculated intakes and UIC (data not shown). Comparison of dietary iodine intake and iodine estimated from UIC and UIE (Table 3) showed that total iodine intake calculated from the 24-h recall as well as the habitual iodine intake by the FFQ were significantly higher than iodine intake estimated from UIE (*p* = 0.17 and *p* = 0.02, respectively). Likewise, iodine intake estimated from UIC was significantly higher than that estimated from UIE (*p* < 0.01). Median (p25–p75) UIC among the supplement users was 120 (80–190) µg/L and 83 (54–120) µg/L among the non-supplement users. 

**Figure 2:**
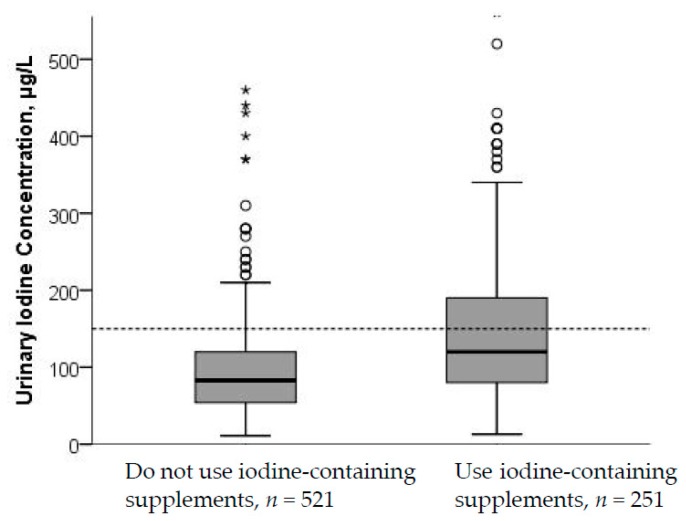
UIC by use of iodine-containing supplements. Five cases with UIC > 500 µg/L were excluded from the figure. The difference in UIC between non-supplement and supplement users was significant (*p* < 0.001; Mann–Whitney U test). The stippled horizontal line marks the epidemiological criteria for assessing adequate iodine nutrition based on median UIC by the WHO [5].

**Figure 3:**
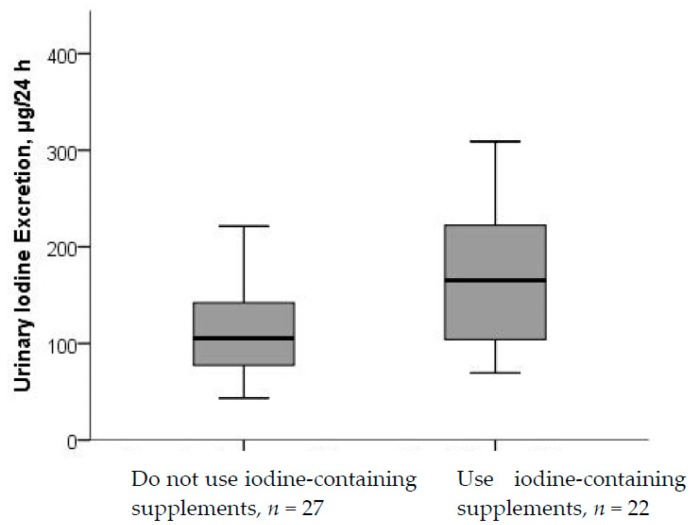
UIE by use of iodine-containing supplements. Differences in UIE between non-supplement and supplement users were significant (*p* < 0.021; Mann–Whitney U test).

Median (p25–p75) UIE among the supplement users was 165 (100–223) µg/24-h and 105 (77–187) µg/24-h among the non-supplement users. Participants who reported use of iodine-containing supplements had higher UIC (*p* < 0.001) than non-supplement users, and, in the sub-study, this was evident also for UIE (*p* = 0.021) (Figure 2 and Figure 3). For UIC, the median values were below the cut-off from WHO of 150 µg/L both among supplement and non-supplement users. In the sub-study, data from the 24-h recall interview showed that the main dietary iodine sources were milk and dairy products, contributing with on average 55% of the daily iodine intake, and seafood contributing with on average 18% of the daily iodine intake. 

Predictors of UIC are shown in Table 4. Maternal age, hypothyroidism, and use of iodine-containing supplements were associated with increased UIC, while gestational weeks and smoking during pregnancy were associated with decreased UIC. These predictors explained 10.5% of the variance in UIC. 

**Figure 4:**
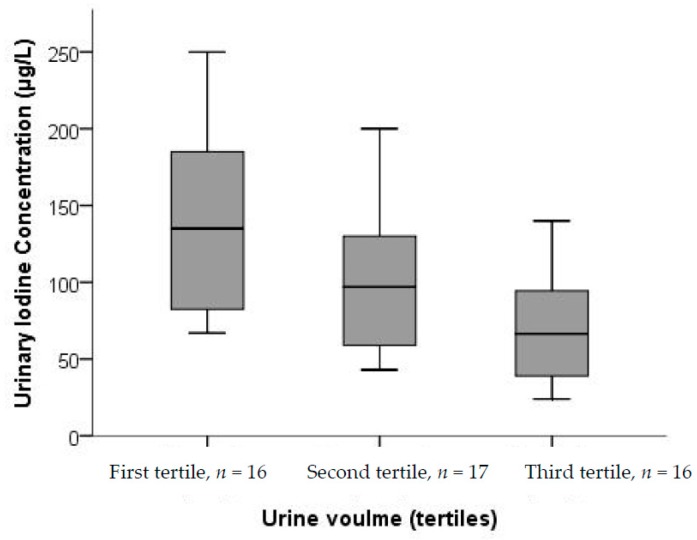
UIC from spot samples in 24-h urine samples by tertiles of total excreted urine volume (*n* = 49). There were significant differences in UIC between the tertiles (*p* = 0.002; Kruskal–Wallis test).

Hydration status is known to influence UIC and in the sub-study we examined UIC by increasing 24-h urine volume and found that UIC in the 24-h urine decreased as urine volume increased (Figure 4). The decrease in UIC by urine volume was significant (*p* = 0.002).

## 4. Discussion

A large proportion of the pregnant women in the Oslo area were mild to moderately iodine deficient. This finding was evident from evaluation of urinary iodine as well as from calculated iodine intake. In utero, severe iodine deficiency causes irreversible damage to the developing brain [6], while the impact of mild to moderate iodine deficiency is less clear. There are indications, however, that mildly decreased maternal thyroid function in pregnancy may result in cognitive delays in the offspring [1,2,3,22,23,24]. These studies show the importance of adequate iodine status during early gestation and emphasize the risk that iodine deficiency can pose to the developing infant, even in countries classified as only mildly iodine deficient. 

Although there is no national screening program for measuring iodine status, Norway has been considered iodine replete for six decades [9,10]. Data from a recent study on iodine status in the Nordic countries found that pregnant and lactating women might be mildly iodine deficient [9]. In the present study, we found a median UIC of 92 μg/L, which is in accordance to previous findings on iodine status among pregnant women in Norway. A Norwegian study in 1008 pregnant women recruited from 2011 to 2012 found that 80% of the pregnant women had UIC below 150 μg/L, with median UIC of 82 μg/L [25]. In the Northern Norway Mother-and-Child Contaminant Cohort Study (MISA), iodine status was measured in 197 pregnant women recruited from 2007 to 2009. A median UIC of 84 μg/L was found and 80% had UIC < 150 μg/L [26]. In the Norwegian Mother and Child Cohort Study (MoBa), 119 participants recruited for a validation study from 2003 to 2004 collected 24-h urine samples. Median UIC was 69 μg/L and 89% had UIC < 150 μg/L [10]. A Norwegian study in 63 non-pregnant women recruited from 1999 to 2001 reported a median UIC of 112 μg/L in Northern Norway and 82 μg/L in Western Norway [27]. According to our knowledge, the present study is the largest and has the most recent data on UIC in Norwegian women (recruited in 2016) and the results add to the evidence that a large proportion of pregnant women have insufficient iodine intake and that iodine nutrition is a public health concern in Norway. Inadequate iodine status has also been reported in other Nordic countries. A cross-sectional study in Denmark from 2012 in 158 pregnant found a median UIC of 119 μg/L [28]. The median UIC was 130 μg/L in iodine-containing supplement users and 76 μg/L in non-supplement users. The median UIC in a Swedish cross-sectional study in 459 pregnant women with urine sampled from 2010 to 2012 was 98 μg/L [29]. 

In the current study, approximately half of the women had a total iodine intake below 160 μg/day, which is the EAR for pregnant women. This strengthens the finding of insufficient iodine intake reflected by low UIC and confirm high probability of iodine inadequacy in this population. Results from the sub-study, in which urinary iodine was measured in 24-h urine samples and participants provided a detailed dietary recall, confirmed the insufficient iodine status observed in the main study. Calculated iodine intake in more than 60,000 pregnant women in MoBa recruited from 2002 to 2008 showed that more than 50% had iodine intake lower than the Nordic recommendations of 175 μg/day [10], and 74% had an estimated intake from food lower than the EAR [1]. Another study in Norwegian pregnant women recruited from 2011 to 2012 (*n* = 833) reported a median iodine intake of 153 μg/day from food and supplements. Use of iodine-containing supplements was reported by 14% of the participants [25]. An increasing number of Norwegians take dietary supplements, and today many multivitamin-mineral supplements contain iodine [9]. The Norwegian guidelines to pregnant women was recently updated to include a recommendation to use of iodine containing supplements (150 µg/day) for women with no or low intake of milk [30]. 

In the present study, 33% of all women reported use iodine-containing supplements, which on average contributed with 175 μg/day to these women’s diet. The median UIC was significantly higher in supplement users than in non-supplement users; 120 μg/day and 83 μg/day, respectively. Similarly, in the MoBa Study, 32% of the pregnant women took iodine-containing supplements which on average contributed with 100 μg/day to these women’s diet [10]. Maternal iodine supplementation in areas of mild-to-moderate iodine deficiency may improve cognitive performance of the offspring, but randomized controlled studies with long-term outcomes are lacking [6].

The iodine requirement increases during pregnancy because of a rise in maternal T4 production to maintain maternal euthyroidism and transfer of thyroid hormones to the fetus, iodine-transfer to the fetus, particularly in late gestation, and a likely increase in renal iodine clearance [31,32]. In Norway, there are few dietary sources of iodine [33] and the individual intake of these food groups varies significantly. In addition, the consumption of seafood, especially lean fish, and milk has declined, especially in the female population [10,34]. In this present study, food groups that contributed most to the iodine intake were milk and milk products (55%) followed by seafood (18%). Even though the participants had a mean daily intake of milk/yoghurt of 3–4 dL, and the majority reported regular consumption of fish, the food sources alone did not provide the amounts of iodine required to meet maternal and fetal needs during pregnancy. In Norway, pregnant women who do not consume or have low intake of dairy and/or seafood, and who do not obtain iodine from supplements are at great risk of inadequate iodine intakes [10,12].

In the multiple regression analysis iodine-containing supplements was the strongest predictor for UIC. The result confirms that iodine-containing supplement use is an important determinant of iodine status and an important iodine source for pregnant women. Maternal age was positively associated with UIC, which has also been found in another study [35]. A potential explanation could be that older women consume more iodine-rich foods [36]. However, this has not been confirmed in Norway. Suffering from hypothyroidism was a strong positive predictor for UIC. Women with hypothyroidism are prescribed a synthetic thyroid hormone (levothyroxine), with iodine, which explains the positive association between hypothyroidism and UIC. A negative borderline significant association between gestational age and UIC was found. One explanation could be an increased iodine transfer to the fetus throughout pregnancy. However, this result is not consistently found in other studies. In a cross-sectional study of pregnant women, UIC increased with gestational age, but the differences between the first, second and third trimester were not statistically significant [37]. In an Austrian study of 246 pregnant women, neither maternal nor gestational age influenced UIC (median UIC: 87 μg/L) [38]. Median UIC in pregnant women in Tasmania declined after elevated excretion of iodine seen in early pregnancy [39]. We found that smoking was negatively associated with UIC. Lower UIC in smokers than in non-smokers was also found in another study in Norway [40]. Tobacco smoke contains thiocyanate, a chemical toxin that accumulates in blood and tissues and has goitrogenic properties [41], but this does not explain why smoking results in lower excretion of iodine. A possible reason for the inverse relationship between smoking and UIC could be related to differences in dietary pattern between smokers and non-smokers. Our results showed no significant association between ethnicity and UIC, contrary to other studies [38,42]. 

In the sub-study, estimated iodine intake from UIE (133 µg/day) were lower than the calculated iodine intakes by in 24-h recall (143 µg/day) and habitual intake (149 µg/day). This might be due to over reporting of food and supplement use or that food iodine concentrations were lower than the values applied in the calculation. Comparison of UIC and urine volume confirmed that hydration status influenced UIC [13,43] and showed that UIE is a more reliable marker of iodine status at the individual level than UIC. However, both habitual iodine intake, 24-h iodine intake and UIC in the 24-h urine correlated with UIE, indicating good conformity between the methods. Collecting 24-h urine samples is a large participant-burden. Furthermore, several 24-h urine collections are needed in order to assess reliable iodine status at the individual level and urine collection is subjected to bias introduced by failure to collect total volume [44]. Results from the sub-study, for which urinary iodine was measured in 24-h urine and who provided a detailed dietary recall as well as habitual food intake, confirmed the insufficient iodine status observed in the main study. 

The major strength of this study is the relatively large sample of pregnant women (*n* = 804) from different parts of Oslo, including 23% with an ethnicity other than Norwegian. UIC from at least 500 individuals are required to define iodine status in a population [8,13]. Secondly, in the sub-study, we assessed both 24-h urinary iodine excretion and collected detailed information on total 24-h and habitual iodine intake. A major weakness of this study is that the women were not randomly selected and therefore the findings cannot be generalized to all pregnant women in Norway. On the other hand, the current study support findings from other studies in other parts of Norway, including the large MoBa study. Selection bias is a concern also in MoBa [45], where the women were better educated and included fewer smokers than the general population of pregnant women. The proportion of highly educated women (≥4 years of University) was significantly higher in our study (40%) than in the rest of the female Norwegian population (8%). Higher education is associated with healthier diet and lifestyle. Despite the high level of education among the participants, insufficient iodine status was evident from both UIC and calculated iodine intakes. The results highlight the often neglected but critical need for routine, periodic monitoring of iodine status in representative population samples, and that national surveys should monitor not only iodine concentrations in school age children, but also include pregnant women as an emerging literature shows that pregnant women may be deficient even where the general population has adequate intake. This implies that iodine insufficiency is present in a larger scale than expected, and it is important the National Health Authorities implement actions immediately. Another limitation of the present study is that a single iodine value was applied to all iodine rich food items (e.g., 130 µg/L) for milk and yoghurt, while the true content is likely subject to seasonal and other variation 

## 5. Conclusions

In conclusion, pregnant women in the Oslo area are mild to moderately iodine deficient. Median UIC and UIE in the sub-study confirmed the finding of insufficient iodine intake in the main study. Approximately half of the women had a total iodine intake below 160 µg/day, which is the EAR for pregnant women. Iodine-containing supplements were the strongest predictor for UIC and an important iodine source for pregnant women, however only one-third reported use of iodine-containing supplements. Comparison of UIC and UIE showed that hydration status influenced UIC and that UIE is a more reliable biomarker for iodine status at the individual level than UIC. In Norway, even with an intake according to the national food-based recommendations, the iodine requirement during pregnancy will not be meet by food only. The current study results underline the importance of implementing actions to improve iodine nutrition in Norway. 

## Figures and Tables

**Table 1:** Sample characteristics of the pregnant women ^a^.

Characteristics	Total Study (*n* = 804)	Main Study (*n* = 755)	Sub-Study (*n* = 49)
Age, years	31.1 ± 4.4	31.1 ± 4.5	30.7 ± 3.5
Pre-pregnancy BMI, kg/m^2^	22.8 (21.0–25.2)	22.8 (20.9–25.3)	22.5 (21.2–24.3)
Gestational weeks ^b^			
1st trimester	28 (3.5)	27 (3.6)	1 (2.0)
2nd trimester	344 (42.8)	329 (43.6)	15 (30.6)
3rd trimester	426 (53.0)	393 (52.1)	33 (67.3)
Parity			
Nulliparous	426 (53.0)	384 (50.9)	42 (85.7)
Primiparous	296 (36.8)	289 (38.3)	7 (14.3)
Multiparous	82 (10.2)	82 (10.8)	0
Country of birth			
Norway	620 (77.1)	579 (76.7)	41 (83.7)
Other	184 (22.9)	176 (23.3)	8 (16.3)
HDI birth country			
Very high HDI	710 (88.3)	663 (87.8)	46 (93.9)
High HDI	37 (4.6)	36 (4.8)	2 (4.1)
Medium HDI	23 (2.9)	23 (3.0)	0
Low HDI	30 (3.8)	30 (4.0)	0
Relationship status			
Cohabiting	429 (53.4)	393 (52.1)	36 (73.5)
Married	347 (43.2)	336 (44.5)	11 (22.4)
Single	19 (2.4)	19 (2.5)	0
Other	9 (1.1)	7 (0.9)	2 (4.1)
Education			
Lower secondary school	25 (3.1)	24 (3.2)	1 (2.0)
Higher secondary school	137 (17.0)	133 (17.6)	4 (8.2)
<4 years of University ^c^	334 (41.5)	317 (42.0)	17 (34.7)
≥4 years of University ^c^	308 (38.3)	281 (37.2)	27 (55.1)
Employment status			
Employed	696 (86.6)	653 (86.5)	43 (87.8)
Stay at home/Unemployed	30 (3.7)	29 (3.8)	1 (2.0)
Student	38 (4.7)	34 (4.5)	4 (8.2)
Other	30 (3.7)	29 (3.8)	1 (2.0)
Iodine supplement use	263 (32.7)	241 (31.9)	22 (44.9)
Smoking during pregnancy	10 (1.2) ^d^	10 (1.3)	0
Self-reported use of dry snuff	10 (1.2) ^d^	10 (1.3)	0
Thyroid disease (self-reported)	38 (4.7)	34 (4.5)	4 (8.1)
Hypothyroidism ^e^	32 (4.0)	29 (3.8)	3 (6.1)
Hyperthyroidism	6 (0.7)	5 (0.7)	1 (2.0)

^a^ Values given in mean ± standard deviation (SD), median (p25–p75) and *n* (%). In the main study: 10 missing from prior body mass index (BMI), 6 missing from gestational age, 4 missing from human development index (HDI), 10 missing from employment status, and 6 missing from thyroid disease. In the sub-study: 2 missing from HDI; ^b^ 1st trimester = 0–12 weeks, 2nd trimester = 13–28 weeks, 3rd trimester = 29 weeks-birth; ^c^ University or University College; ^d^ Daily amounts ranged from 1–10 cigarettes, both occasionally and daily use; ^e^ Treated with synthetic T4.

**Table 2:** Urinary iodine concentration (UIC) by trimester in spot samples (*n* = 777) ^a^, and UIC, urine volume and urinary iodine excretion (UIE) in 24 h urine samples in the sub-study (*n* = 49).

	UIC (*n* = 777)				
UIC (μg/L)	Median	p25–p75	Mean	SD	Min, Max
All trimesters (*n* = 777)	92	59–140	114	86	11, 860
1st Trimester (*n* = 26)	92	43–173	115	75	23, 280
2nd Trimester (*n* = 332)	96	64–140	119	94	14, 860
3rd Trimester (*n* = 413)	91	58–130	110	80	11, 660
	**UIE (*n* = 49)**				
UIC in 24-h urine (µg/L)	91	61–140	103	54	24, 250
Urine volume (L/24 h)	1.4	1.1–1.8	1.5	0.5	0.5, 3.2
UIE (µg/24 h)	120	83–181	136	64	43, 309

^a^ Six had missing information for gestational age. No significant differences between trimesters (*p* = 0.381), tested by Kruskal–Wallis test.

**Table 3:** Calculated iodine intake from food and from food and supplements reported from 24-h recall of iodine-rich foods in the main study and habitual intake and 24-h recall in the sub-study.

	Main Study (*n* = 755)				
Iodine Intake (µg/day)	Median	p25–p75	Mean	SD	Min, Max
24-h intake from food ^a^	110	70–150	121	67	30, 667
24-h total intake ^b,§^	148	86–251	175	105	30, 689
Estimated intake from UIC	145	89–214	176	133	16, 1183
	**Sub-Study (*n* = 49)**				
24-h intake from food	114	78–149	128	75	18, 403
24-h total intake ^c,§§^	143	101–289	188	106	18, 403
Habitual intake from food	117	95–147	122	37	50, 206
Habitual total intake ^d,§§^	149	109–268	182	90	56, 361
Estimated intake from UIC ^e^	157	103–257	193	122	103, 257
Estimated intake from UIE ^e,f^	133	92–201	151	71	48, 343

Differences tested with Wilcoxon Signed Ranks test; ^a^ Iodine intake from iodine-rich foods; ^b^ Iodine intake from iodine-rich foods and supplements; ^c^ Iodine intake from food and supplements; ^d^ Iodine intake from food and supplements; ^e^ Estimated iodine intake from UIC = UIC (μg/L) × 0.0235 × bodyweight (kg) [19]; ^f^ Estimated iodine intake from UIE given 90% excretion of ingested iodine = UIE (µg/24-h) × 100/90; ^§^ Total 24-h iodine intake did not differ from iodine intake estimated from UIC (*p* = 0.2; Wilcoxon’s signed rank test); ^§§^ Total 24-h iodine intake, total habitual iodine intake, and iodine intake estimated from UIC were all significantly higher than iodine intake estimated from UIE (*p* < 0.05; Wilcoxon’s signed rank test).

**Table 4:** Predictors of urinary iodine concentration (UIC) in pregnant women in Oslo (*n* = 777), with UIC ^a^ (μg/L) as dependent variable.

Predictor Variables	Unadjusted Coefficient (95% CI)	*p*	Adjusted Coefficient (95% CI)	*p*	Stand Beta
Constant			4.181 (3.849, 4.513)	<0.001	
Maternal age	0.015 (0.005, 0.025)	0.004	0.013 (0.003, 0.022)	0.011	0.088
Gestational weeks	−0.004 (−0.010, 0.001)	0.139	−0.007 (−0.012, −0.001)	0.019	−0.081
Hypothyroidism ^b^	0.291 (0.057, 0.525)	0.015	0.313 (0.090, 0.536)	<0.001	0.289
24-h iodine Suppl ^c^	0.002 (0.001, 0.002)	<0.001	0.002 (0.002, 0.003)	0.006	0.094
Smoking ^d^	−0.529 (−0.951, −0.108)	0.014	−0.442 (−0.869, −0.016)	0.042	−0.070
*R*^2^				0.105	

^a^ UIC log transformed, 6 missing from gestational age and hypothyroidism, and 10 missing in the adjusted model due to missing cases in independent the variables mentioned, (95% CI = 95% confidence interval); ^b^ Self-reported hypothyroidism (0 = no, 1 = yes); ^c^ Mean daily intake of iodine from supplements (habitual intake); ^d^ Categories for smoking during pregnancy (0 = no, 1 = yes).
